# Identification and selection of reference genes for analysis of gene expression by quantitative real-time PCR in the euhalophyte *Suaeda altissima* (L.) Pall.

**DOI:** 10.1080/19420889.2024.2372313

**Published:** 2024-07-08

**Authors:** Dmitrii E. Khramov, Olga I. Nedelyaeva, Alena O. Konoshenkova, Vadim S. Volkov, Yurii V. Balnokin

**Affiliations:** a Laboratory of Ion Transport and Salinity Tolerance, K.A. Timiryazev Institute of Plant Physiology of the Russian Academy of Sciences, Moscow, Russia; b Department of Plant Sciences, College of Agricultural and Environmental Sciences, University of California, Davis, CA, USA

**Keywords:** Halophyte, nitrate deficiency, *SaNPF6.3* nitrate transporter gene, reference genes, *Suaeda altissima*, salinity

## Abstract

Сoding sequences of seven housekeeping genes: actin *SaACT7*, ubiquitin-conjugating protein *SaUBC10*, glyceraldehyde-3-phosphate dehydrogenase *SaGAPDH*, protein of the large subunit of ribosomes *SaL2*, α-tubulin *SaTUA*, translation elongation factor *SaeEF1α*, and protein phosphatase *SaPP2A* were identified as candidate reference genes for expression analysis of target genes in the extremely salt tolerant plant *Suaeda altissima* (L.) Pall. The expression profiles of the genes differed. *SaACT7* and *SaeEF1α* demonstrated the highest expression levels, while the lowest expression levels were found for *SaPP2A* and *SaTUA*. *SaPP2A* and *SaeEF1α* genes were the most stably expressed at different steady-state salinity levels and different nitrate concentrations in nutrient solutions (NSs). *SaL2*, *SaPP2A*, and *SaeEF1α* genes showed the greatest stability of expression when nitrate was added to nutrient solution of plants grown under conditions of nitrate deficiency. Less constant expression was demonstrated in this experiment by *SaACT7* and *SaTUA*. *SaL2, SaACT7, SaeEF1α*, and *SaUBC10* genes showed the smallest expression changes under salt shock. To validate the use of the most stably expressed genes for normalization of gene expression, we checked them as reference genes to study the expression of the nitrate transporter gene *SaNPF6.3* in *S. altissima* roots under conditions of different salinity and different nitrate supply.

## Introduction

The genus *Suaeda* Forrsk. belongs to the family Amaranthaceae (subfamily Chenopodioideae). Species of this genus are mostly herbaceous succulent plants and are typical euhalophytes [1,2]. Due to the ability of *Suaeda* species to populate highly saline soils and the recent progress in *Suaeda* transcriptomics and genomics, members of this genus have become model plants for investigating mechanisms underlying salt tolerance [2,3]. The habitat of *Suaeda altissima*, to which this paper is devoted, is often coastal solonchaks (https://ehaloph.uc.pt/showplant/11496, cited 2023 Nov 19). This species is widespread in the European part of Russia, the Caucasus, Crimea, Western Siberia and Central Asia [4] (https://ehaloph.uc.pt/showplant/11496, cited 2023 Nov 19). In hydroponics, *S. altissima* is able to grow in nutrient solutions (NSs) both in the absence and presence of NaCl at concentrations up to 1 M, while 250 mM NaCl stimulates its growth [5]. Due to these properties and the ability to grow under hydroponic conditions allowing strict control of the NS ionic composition, *S. altissima* is used as a model to study the Na^+^- and Cl^–^-homeostasis mechanisms in salt tolerant plants [6–11]. *S. altissima* is also of interest from a practical point of view. In arid lands, this species may be used in artificial phytocenosis as a fodder plant [12], as well as for phytoremediation of saline and degraded territories subjected to unsustainable exploitation and natural desertification process. Being a salt-accumulating halophyte, *S. altissima* is a promising species for the extraction of heavy metals from soil [13]. Attempts have been made to use the closely related species *S. salsa* for soil desalinization [14,15].

In plants inhabiting saline soils, uptake and further transport of K^+^ and NO_3_^–^, both essential for life, occurs in competition with Na^+^ and Cl^–^, which may be toxic [16–20]. At high NaCl concentrations in the environment, ion-transporting proteins of halophytes, unlike those of glycophytes, cope with uptake and distribution of the essential ions throughout the plant. The halophytic ion channels and transporters are assumed to differ from their glycophytic orthologs by primary structure and, accordingly, ionic selectivity and kinetic characteristics which allows halophytes to take up essential elements under saline conditions [18–21].

The studies on the primary structure and functional features of ion-transporting proteins from *Suaeda* species is an important facet of investigations of the mechanisms underlying salt tolerance [22–27]. The development of nucleotide sequencing techniques has allowed the analysis of transcriptomes and genomes of members of the *Suaeda* genus [28–30]. Arrays of short RNA reads were obtained from *S. fruticosa* [28] and *S. glauca* [29], analysis of which, based on the *de novo* transcriptome assembly method, allowed identification of the coding sequences for a number of genes in *S. altissima* [23,25–27,31]. Genomic DNA sequences were assembled, and a large number of genes in *S. glauca* [32,33] and *S. aralocaspica* [34,35] involved in various metabolic processes and physiological functions were annotated.

Understanding the mechanisms responsible for salt tolerance requires the discovery of their specific molecular determinants (comprehensively reviewed in [36–38]). One approach to identifying molecular determinants is to examine the expression of candidate genes for involvement in the formation of a given trait. Quantitative real-time polymerase chain reaction after reverse transcription (RT-qPCR) is widely used to study the expression of genes of interest, which allows quantification of their mRNA content in tissues. However, in order to obtain correct results and their proper interpretation, it is necessary to use reference genes, the expression level of which does not noticeably fluctuate under changing experimental conditions. The reliability of reference genes, i.e. the stability of their expression is species-specific and can change significantly under biotic and abiotic environmental factors. Therefore, the selection of reference genes should be carried out for each species separately and in accordance with the objectives of the experiment. Particularly, the expression of a reference gene should be maintained at the same level in control and experimental samples when elucidating the effects of NaCl on the expression of genes of interest.

A large number of experiments have been done to identify the stability of expression of reference genes in model *(Arabidopsis thaliana, Nicotiana tabacum*) and cultivated (e.g. *Solanum tuberosum, Pisum sativum, Oryza sativa, Glycine max, Hordeum vulgare, Triticum aestivum, Vitis vinifera, Prunus persica, Fragaria spp, Pyrus bretschneideri*) plants (a comprehensive list of plant species and the corresponding references is not provided here due to the limitations of space). Less work has been done for wild plants of arid climates compared to model and cultivated; especially little is known for halophytes.

To investigate the effects of NaCl and salt tolerance mechanisms in *Suaeda altissima*, the reference genes suggested for the halophytes *Salicornia europaea* [39], *Suaeda aralocaspica* [40], *Hordeum brevisubulatum* [41] and *Suaeda glauca* [42] were checked. A set of six genes was considered for *S. aralocaspica* as candidate reference genes: *β-TUB* and *GAPDH* were shown to be expressed most stably at different stages of plant development, *ACTIN* exhibited the highest expression stability under abiotic stresses of different types, and expression of *18S rRNA* and *28S rRNA*, genes of ribosomal RNAs was the least stable under these conditions [40]. A set of ten genes was presented for *S. glauca*. The *PP2A* and *TUA5* genes showed the highest expression stability in different tissues under different salinity regimes, the *DREB1D* gene was expressed the least stably [42]. In the present work, we selected seven genes involved in different vital cellular processes and physiological functions (hereafter candidate genes) and tested them for application as reference genes in studies of high salinity and nitrate deficiency effects on target gene expression in the euhalophyte *Suaeda altissima*. They included the housekeeping genes [43] encoding the proteins of the cytoskeleton, protein synthesis/degradation machineries and important biochemical processes: SaACT7 actin, SaUBC10 ubiquitin-conjugating protein, SaGAPDH glyceraldehyde-3-phosphate dehydrogenase, SaL2 ribosome large subunit protein, SaTUA α-tubulin, SaeEF1α translation elongation factor, and SaPP2A protein phosphatase. Among them, the most stably expressed genes were selected under conditions of salt stress and nitrate deficiency and tested for suitability to use as reference genes for *S. altissima* when studying the expression of the nitrate transporter gene *SaNPF6.3*.

## Materials and methods

### Plant material

*S. altissima* (L.) Pall. was cultivated in hydroponics under controlled conditions, as it was described previously [26]. Briefly, the plants were illuminated with high pressure sodium lamps DNaZ_400 (Re- flux, Russia) with a photoperiod of 12 h/12 h (day/night) and a light intensity of 300 µmol photons/(m^−2^ s^−1^) and grown in Robinson-Downton medium [44]. The concentration of NO_3_^–^ was changed from 5 mM to 15 mM (standard concentration) or 0.5 mM (nitrate deficiency). In the case of long-term salinity (250 mM or 750 mM), NaCl was added to the NS of 28-day-old plants in increment of 50 mM (first 5 days) and 100 mM (next 5 days). In order to simulate salt shock, NaCl was added to the NS of 45-day-old plants to produce a concentration of 250 mM in one-step. To investigate the stability of candidate gene expression, 45-day-old plants grown (1) in the NS under long-term salinity (250 mM, 750 mM NaCl) or in the absence of NaCl at the background of standard or low NO_3_^–^, (2) in the absence of NaCl at low NO_3_^–^ followed by one-step addition of NO_3_^–^ to a final concentration of 5 mM, and (3) in the absence of NaCl at low NO_3_^–^ followed by one-step addition of NaCl in a concentration of 250 mM were used.

### Extraction of total RNA from plant material and synthesis of the first strand cDNA

Total RNA was isolated by the hot phenolic method, as described earlier [26] and was used as a template for the first-strand cDNA synthesis. For amplification of the 3´- and 5´- end transcript sequences by the Step-Out RACE method, the first-strand cDNA was synthesized on a total RNA template using MINT revertase (Evrogen, Russia). To obtain full-length cDNAs and quantify the representation of the gene transcripts in *S. altissima* organs, first-strand cDNA synthesis was performed on a total RNA template using (dT)_15_ primer and MMLV revertase (Evrogen, Russia).

### Cloning the coding sequences of candidate reference genes

*In silico* search for sequences homologous to *AtACT7* (AT5G09810, NP_196543.1), *AteEF1α* (AT1G07940, NP_001030993.1), *AtGAPDH* (AT1G13440.1, NP_172801.1), *AtUBC10* (AT5G53300.1, NP_001190528.1), *AtL2* (AT2G18020.1, NP_179393.1), *AtTUA* (AT5G19780.1, NP_197478.1), and *AtPP2A* (AT1G69960, NP_177154.1) was performed using the earlier transcriptome of *Suaeda fruticosa* (L.). Forssk, a closely related species *S. altissima*, *de novo* assembled by us [31]. Further on, the contigs of the assembled *S. fruticosa* transcriptome were translated into amino acid sequences. In the data obtained, the search of the sequences that were most similar to *A. thaliana* proteins AtACT7, AteEF1α, AtGAPDH, AtUBC10, AtL2, AtTUA, and AtPP2A was accomplished using the BLAST program (https://blast.ncbi.nlm.nih.gov/Blast.cgi). The primer pairs (Supplementary Material, Table S1) were matched using the contigs identified in the assembled *S. fruticosa* transcriptome. The full-size coding sequences of the homologous *S. altissima* genes were then amplified from *S. altissima* cDNA template with these primers using Encyclo-polymerase (Eurogen, Russia). PCR products were cloned into pAL2-T (Evrogen, Russia) or pTZ57R/T (Thermo Fisher, USA) vector, sequenced and deposited in GenBank: *SaACT7* (MK615596), *SaeEF1α* (MN076325), *SaGAPDH* (OP752353), *SaL2* (OP752354), *SaPP2A* (OP752355), *SaUBC10* (OP752356), *SaTUA* (OP752357) (Supplementary materials, Table S2).

### Quantitative analysis of transcripts for candidate reference genes in S. altissima organs

**Figure 1.**
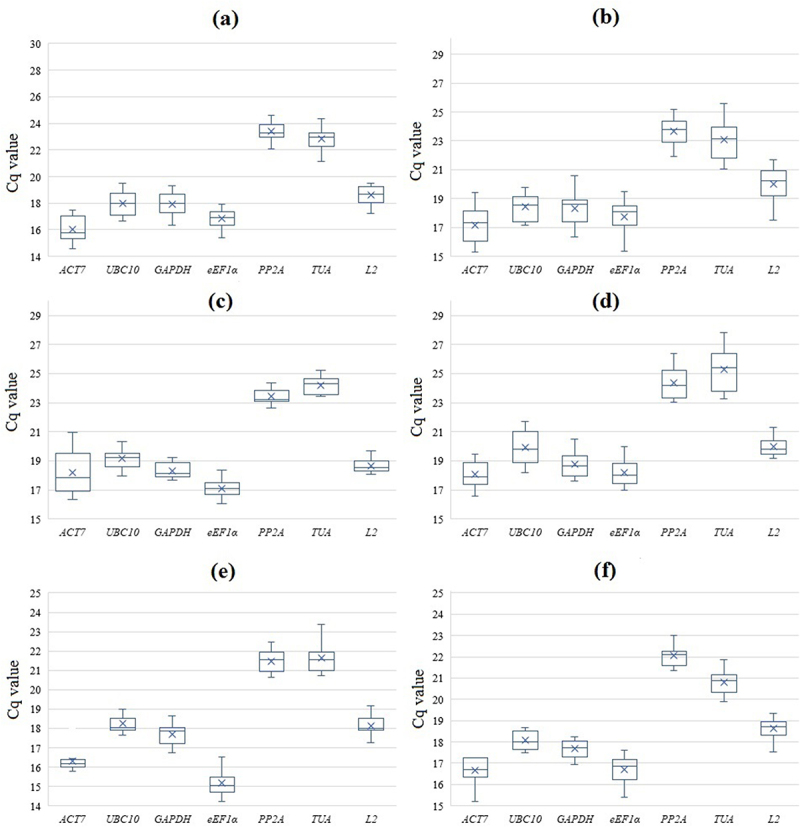
Expression profiles of *S. altissima* candidate reference genes as box plots. Raw Cq values are represented on the ordinate axis. The box shows the 25th and 75th percentiles of data sampling, and the whiskers indicate the maximum and minimum values ignoring drop-out ones. The median and mean are indicated by a cross and a cross line, respectively. (a, b) – long-term salinity (0, 250, 750 мМ NaCl); (c, d) – one-step increase in the concentration of nitrate in the NS from 0.5 to 5 mM; (e, f) – salt shock (250 мМ NaCl); (a, c, e) – roots, (b, d, f) – leaves.

The quantitative analysis of transcripts was performed by RT-qPCR using a LightCycler® 96 System (Roche Diagnostics Corporation, Indianapolis, USA). A reaction mixture with intercalating dye SYBR Green I (Evrogen, Moscow, Russia) was used. The amplification program was designed according to the manufacturer’s recommendations. The primers for the candidate genes *SaeEF1α*, *SaACT7*, *SaGAPDH*, *SaTUA*, *SaUBC10*, *SaL2*, *SaPP2A*, and for the nitrate transporter gene *SaNPF6.3* (Supplementary Material, Table. S1, Figure 1) were selected using Light Cycler Probe Design Software 2.0 (Roche, USA).

The results are based on three biological replicates, each biological replicate was performed in 3 analytical replicates. The material for obtaining one biological replicate was a mixture of finely chopped organs (roots or leaves) collected from 5–6 plants grown in hydroponics.

### Determination of candidate gene expression stability using geNorm algorithm

To compare the expression stability of candidate genes based on the geNorm algorithm, *M* values, normalization factors (NF) and pairwise variations (V_n_/_n + 1_) of normalization factors were calculated for each candidate gene according to [45] (https://genorm.cmgg.be/). The *M* value specifies the stability of gene expression. The threshold value of *M* value is 1.5; if the value of M is greater than 1.5, the gene expression is unstable and the use of this gene as a reference gene is excluded. The smaller the value of M, the greater is the stability of the gene expression. The minimum number of reference genes sufficient to normalize target gene expression in RT-qPCR reliably was determined by calculating pairwise variations in normalization factors. The threshold value of V_n_/_n + 1_ is 0.15, when V_n_/_n + 1_ ˂ 0.15, the inclusion of one more gene in the stability assay does not affect normalization. Sequential inclusion of genes in the analysis should be continued until the V_n_/_n + 1_ values are reduced to the threshold level.

### Determination of candidate gene expression stability using NormFinder algorithm

Determination of expression stability using NormFinder algorithm was performed according to [46] (https://moma.dk/normfinder-software). This algorithm is based on the calculation of intra- and intergroup variations. Genes with the lowest expression stability values are expressed the most stably.

### Determination of candidate gene expression stability using BestKeeper software tool

Determination of expression stability using BestKeeper software was performed in accordance with [47] (https://www.gene-quantification.de/bestkeeper.html). Standard deviation (SD) and coefficient of variation (CV) are the parameters of the BestKeeper software tool that determine the stability of gene expression. The smaller the SD and CV values, the more stable the expression of reference genes. If the SD value of a gene is greater than 1, the gene is excluded from normalization.

### Normalization of the nitrate transporter gene SaNPF6.3 expression

To validate the suitability of the selected genes as reference genes for *S. altissima*, we examined expression of the nitrate transporter gene *SaNPF6.3* cloned previously (NCBI, GenBank OQ330855), under different conditions and evaluated its expression profiles. For this purpose, the normalization factor was first calculated as a geometric mean of expression levels of selected reference genes according to the equation:

$$\mathrm{NF}\;=\sqrt[{n}]{2^{\mathrm{Cq}1}\cdot 2^{\mathrm{Cq}2}\cdot \ldots \cdot 2^{\mathrm{Cqn}}},$$

where NF is the normalization factor; Сq1, Сq2, … , Сqn are the threshold levels of reference genes with the most stable expression [45]. Then, the relative level of expression of *SaNPF6.3* transcripts was determined according to the formula: NF/2^CqSaNRT1.1^, where Cq_SaNRT1.1_ is the value of *SaNPF6.3* expression threshold level. The values of *SaNPF6.3* transcript abundances are presented as means ± SD, the values for the control conditions are taken as a unit. One-factor analysis of variance was performed, *P ≤.05; **P ≤.01 based on 3–5 biological replicates.

## Results

Seven genes, *SaACT7*, *SaeEF1α*, *SaGAPDH*, *SaL2*, *SaPP2A*, *SaUBC10*, *SaTUA* were pre-selected so that suitable reference genes could then be selected from these genes to investigate salinity and nitrate deficiency effects on the expression of target genes in *S. altissima*. The coding sequences of the candidate genes were cloned and translated into amino acid sequences. The latter were highly conservative. Alignment with the orthologs from *A. thaliana* showed that the proportion of identical amino acid residues for each of the seven proteins exceeded 90% (Supplementary materials, Table S2). All candidate genes were expressed at different stages of plant development. Expression occurred in 21- and 45-day-old plants in cotyledons, leaves, roots, and stems, as well as in 60-day-old plants in flowers (Supplementary materials, Table S3) at a relatively stable levels indicating the importance of all these candidate genes for *S. altissima*. It is interesting to mention that the threshold values of cycle numbers (Cq values) were higher (indicating lower abundance of transcripts) for all the genes in flowers of 60-day-old plants (Supplementary materials, Table S3).

The stability of candidate gene expression was investigated using three statistical algorithms and software tools (geNorm, NormFinder, and BestKeeper) for different NaCl and nitrate concentrations in the NS. The threshold values of cycle numbers (Cq values) in RT-qPCRs varied significantly among the different genes. The lowest Cq values were for actin and elongation factor, while the highest Cq values were for protein phosphatase and tubulin. The threshold values of cycle numbers ranged from 14.61 (*ACT7*) to 27.84 (*PP2A*) (Figure 1, Supplementary materials, Table S3). Overall, the profiles of candidate gene expression patterns were similar in the three experimental designs (see Material and methods), as well as in roots and leaves, although differences in gene expression levels were observed (Figure 1). The levels of candidate gene transcript abundance under all experimental conditions decreased in the series *ACT7* ≥ *eEF1α* ˃ *GAPDH* ˃ *UBC10* ˃ *L2* ˃ *TUA* ≥ *PP2A*. We concluded that the Cq values are rather similar in the replicates. Most of Cq values in the replicates for the majority of candidate genes were located near the median (Figure 1).

### geNorm

**Figure 2.**
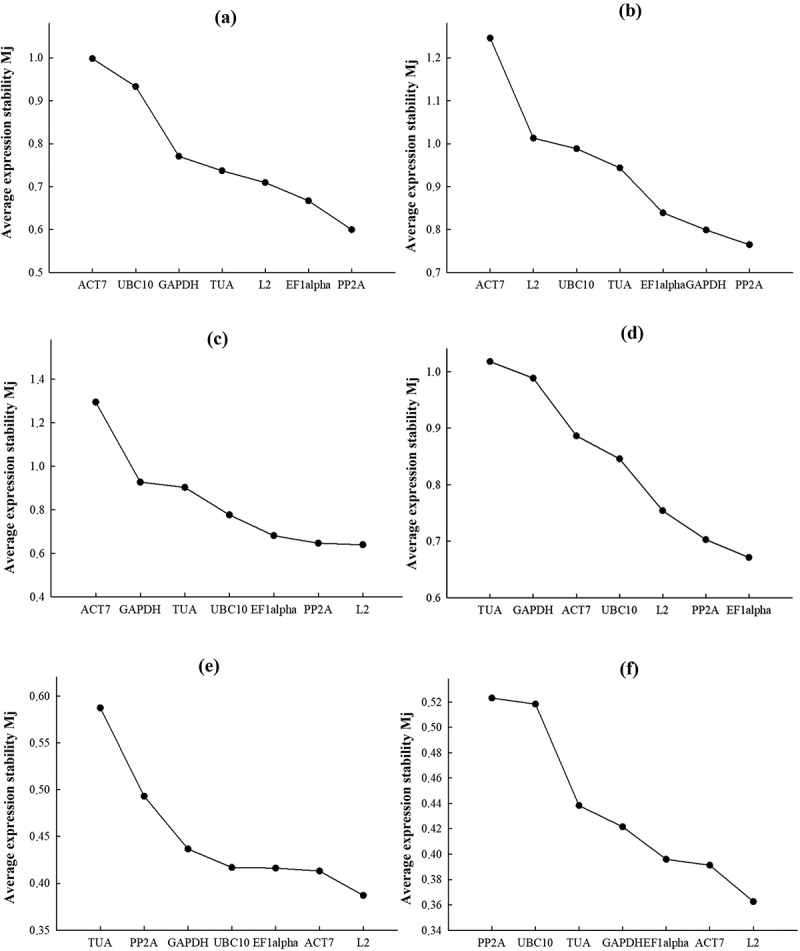
Stability of candidate reference gene expression using geNorm algorithm. Candidate reference gene expression stability values, Mj, calculated using geNorm algorithm in *S. altissima* organs. Mj values for each reference gene are ranked in descending order (from left to right), corresponding to increasing stability of candidate reference gene expression. (a, b) – long-term salinity (0, 250, 750 mM NaCl); (c, d) – one-step increase in the concentration of nitrate in the NS from 0.5 to 5 mM; (e, f) – salt shock (250 mM NaCl); (a, c, e) – roots; (b, d, f) – leaves.

**Figure 3.**
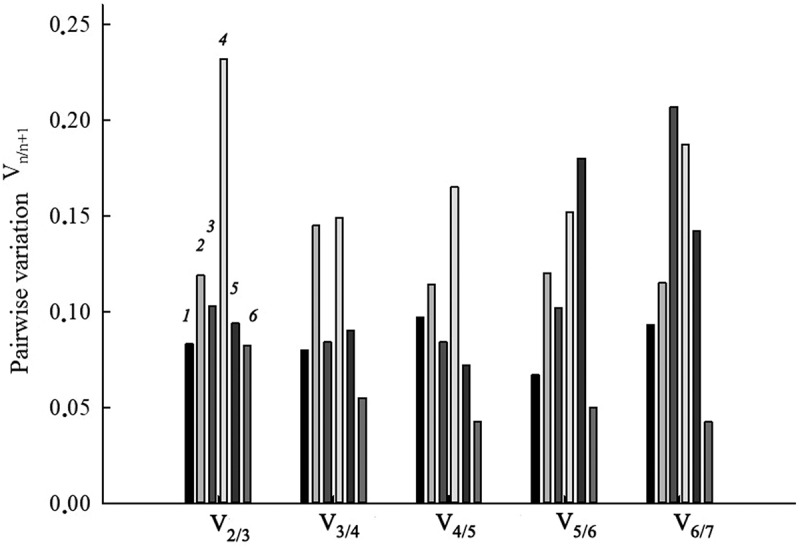
Determination of the optimal number of reference genes for target gene expression normalization under conditions of salt stress and nitrate deficiency in *S. altissima* organs based on analysis of pairwise variation values (V_n_/_n +1_) of normalization factors of the candidate reference genes. *1* – long-term salinity (0, 250, 750 mM NaCl), roots; *2* –long-term salinity (0, 250, 750 mM NaCl), leaves; *3* – one-step increase in the concentration of nitrate in the NS from 0.5 to 5 mM, roots; *4* – one-step increase in the concentration of nitrate in the NS from 0.5 to 5 mM, leaves; *5* – salt shock (250 mM NaCl), roots; *6* – salt shock (250 mM NaCl), leaves.

The analysis of candidate gene expression stability using the geNorm program algorithm (Figures 2 and 3) is based on the comparison of Mj values of candidate genes [45]. Mj represents the average standard deviation of a logarithm-transformed expression level set of a candidate gene j relative to other candidate genes. The larger the Mj value, the less stable is the expression of gene j. The best reference gene corresponds to the minimum value of Mj. The stability of candidate gene expression according to geNorm analysis varied significantly; however, for all candidate genes in each experiment Mj values was below the threshold level of 1.5 ranging from 1.39 (*ACT7*) to 0.36 (*L2*) (Figure 2).

Under long-term salinity conditions at different nitrate availability in the NS, *PP2A*˃*eEF1α*˃*L2*˃*TUA* were the more stably expressed genes in roots and *PP2A*˃*GAPDH*˃*eEF1α*˃*TUA* in leaves (Figure 2a,b). When the nitrate concentration in nitrate-deficient medium (0.5 mM) was raised to 5 mM, a more stable expression was observed for *L2* ˃*PP2A* ˃*eEF1α*˃ *UBC10* genes in roots and *eEF1α*˃*PP2A*˃*L2*˃*UBC10* genes in leaves (Figure 2c,d). In the salt shock experiments, the stability of candidate gene expression decreased in *L2>ACT7>>eEF1α>UBC10* (roots) and *L2>ACT7>>eEF1α>GAPDH* (leaves) series (Figure 2e,f). The least stably expressed genes according to the geNorm algorithm were *ACT7* under long-term salinity conditions at different nitrate availability in the NS; *ACT7*, *GAPDH*, and *TUA* when nitrate was added to nitrate-deficient medium; *TUA* in roots and *PP2A* in leaves in the salt shock experiment (Figure 2).

The optimal number of reference genes required for accurate normalization under the conditions of each experiment was determined by calculating the pairwise variation values (V_n_/_n + 1_) of normalization factors (NFs) in roots and leaves (Figure 3). According to the analysis in the long-term salinity and salt shock experiments, the use of the two most stably expressed genes was sufficient to reliably normalize the expression in both roots and leaves (Figure 3), because the V_2/3_ value under these conditions was below than the threshold level of 0.15. In the experiment with nitrate addition to nitrate-deficient medium, the use of two genes was also sufficient to normalize expression in roots, but three genes were required to normalize expression in leaves (V_2/3_ = 0.232; V_3/4_ = 0.149).

### NormFinder

The lower the value of the expression stability index calculated according to NormFinder’s algorithm [46], the higher is the expression stability (Table 1). In the long-term salinity experiments, *PP2A*˃*eEF1α*˃*L2*˃*GAPDH* genes were more stably expressed in roots and *PP2A*˃*GAPDH*˃*eEF1α*˃*TUA* genes in leaves. In the experiments with nitrate addition to nitrate-deficient medium, higher expression stability was observed for the genes *L2*˃*PP2A*˃*SaeEF1α*˃*TUA* in roots and for *eEF1α*˃*PP2A*˃*L2*˃*UBC10* in leaves. In the case of salt shock, the more stably expressed genes were *L2*˃*UBC10*˃*eEF1α*˃*ACT7* in roots and *L2*˃*TUA*˃*GAPDH*˃*UBC10* in leaves. In the salt-shock experiments, relatively small values of the expression stability index were found for all candidate genes in leaves (Table 1) indicating their stable expression in this organ. Overall, the assessment of candidate gene expression stability for *S. altissima* using the NormFinder algorithm is similar to that according to geNorm program.

**Table 1.** Values of the expression stability index of *S. altissima* candidate reference genes calculated using NormFinder program algorithm.

Gene	Long-term salinity		Nitrate addition		Salt shock	
	root	leaf	root	leaf	root	leaf
*SaACT7*	0.325	0.526	0.607	0.434	**0.102**	0.011
*SaeEF1α*	**0.161**	**0.311**	**0.259**	**0.186**	**0.097**	0.010
*SaGAPDH*	**0.204**	**0.281**	0.371	0.427	0.109	**0.008**
*SaL2*	**0.201**	0.438	**0.189**	**0.274**	**0.077**	**0.002**
*SaPP2A*	**0.103**	**0.232**	**0.239**	**0.192**	0.133	0.010
*SaTUA*	0.236	**0.407**	**0.264**	0.504	0.203	**0.006**
*SaUBC10*	0.358	0.436	0.346	**0.304**	**0.086**	**0.009**

The lowest half of values for the stability index of the most stably expressed genes are given in bold.

### BestKeeper

According to the algorithm of the BestKeeper program, the stability of candidate gene expression is assessed by the values of the standard deviation of gene expression levels (SD) and coefficients of their variation (CV) [47]. The genes with the lowest SD and CV values are characterized by the most stable expression, the genes with SD values greater than 1 are referred to as unstably expressed genes and are excluded from consideration. In the experiments with long-term salinity, *PP2A* ˃ *L2* ˃˃*eEF1α*˃*GAPDH* were more stably expressed genes in roots and *PP2A ˃UBC10˃GAPDH˃eEF1α* in leaves (Table 2). In the case of nitrate addition to nitrate-deficient medium, *L2*˃*PP2A*˃*eEF1α*˃*GAPDH* genes were expressed more stably in roots and *L2*˃*eEF1α*˃*GAPDH*˃*GAPDH*˃*ACT7* genes in leaves. Under salt shock conditions, *ACT7*˃*L2*˃*UBC10*˃*PP2A* genes in roots and the *L2*˃*UBC10*˃*GAPDH* ˃*PP2A* genes in leaves displayed little fluctuation of expression. *ACT7* and *TUA* were characterized by the highest instability at long-term salinity and when nitrate was added to the NS; SD values for *ACT7* and *TUA* exceeded unity under these experimental conditions.

**Table 2.** Expression stability indices (SD, CV) of *S. altissima* candidate reference genes calculated using BestKeeper program.

Gene	Long-term salinity				Nitrate addition				Salt shock			
	root		leaf		root		leaf		root		leaf	
	SD	CV	SD	CV	SD	CV	SD	CV	SD	CV	SD	CV
*SaACT7*	0.81	5.03	1.00	5.83	1.21	6.66	**0.79**	**4.39**	**0.36**	**2.19**	0.43	2.62
*SaeEF1α*	**0.59**	**3.48**	**0.89**	**5.02**	**0.43**	**2.53**	**0.69**	**3.81**	0.50	3.30	0.46	2.80
*SaGAPDH*	**0.68**	**3.80**	**0.82**	**4.50**	**0.44**	**2.42**	**0.71**	**3.77**	0.43	2.41	**0.35**	**1.97**
*SaL2*	**0.52**	**2.79**	0.90	4.52	**0.36**	**1.92**	**0.54**	**2.71**	**0.37**	**2.02**	**0.33**	**1.78**
*SaPP2A*	**0.51**	**2.19**	**0.74**	**3.15**	**0.43**	**1.83**	0.89	3.66	**0.42**	**1.94**	**0.36**	**1.62**
*SaTUA*	0.74	3.25	1.01	4.40	0.52	2.17	1.22	4.82	0.56	2.58	0.47	2.27
*SaUBC10*	0.69	3.86	**0.74**	**4.05**	0.53	2.76	0.99	4.98	**0.40**	**2.19**	**0.35**	**1.96**

The lowest half of values for standard deviation (SD) and (CV) values for the most stably expressed genes are given in bold.

Summarizing the results of calculations using these three algorithms, we can conclude that the more stably expressed candidate genes in the organs of *S. altissima* were (1) *PP2A* and *eEF1α* under long-term salinity at different nitrate concentrations in the NS; (2) *PP2A*, *eEF1α*, *L2* when nitrate was added to the nitrate-deficient NS; and (3) *L2*, *ACT7*, *eEF1α*, and *UBC10* under condition of salt shock.

### Expression of the nitrate transporter gene SaNPF6.3

**Figure 4.**
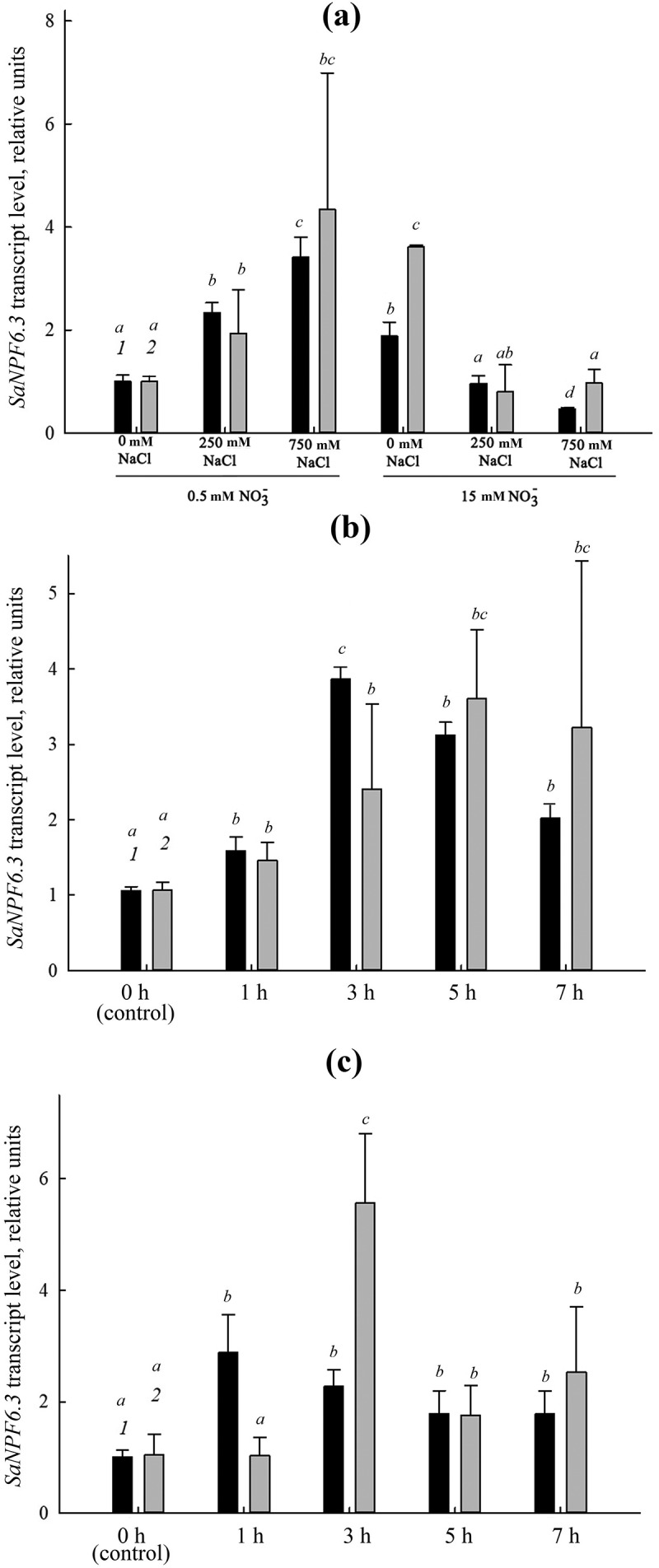
Relative representation of transcripts of the nitrate transporter gene *SaNPF6.3* in *S. altissima* roots under: (a) long-term salinity (0, 250, 750 mM NaCl) at different nitrate availability (0.5 or 15 mM NO_3_^–^) in the NS relative to the normalization factor NF *(eEF1+PP2A)* (black bars, 1) and *ACT7* (gray bars, 2); (b) salt shock (250 mM NaCl) at low nitrate concentration in the NS (0. 5 mM) relative to NF *(ACT7+L2)* (black bars, 1) and *TUA* (gray bars, 2); (c) one-step increase in nitrate concentration in the NS from 0.5 to 5 mM relative to NF *(eEF1+PP2A)* (black bars, 1) and *ACT7* (gray bars, 2). Different letters indicate significant difference (*p*-value <.05).

Among the candidate genes, the more stably expressed genes were used as reference genes to calculate normalization factors and to investigate the relative abundance of transcripts in *SaNPF6.3*, a nitrate transporter gene of the *NPF*/*NRT1* family, in roots (Figure 4). The product of this gene is involved in nitrate uptake by roots and probably in chloride transport [27]. *SaNPF6.3* expression in roots depended on NaCl and nitrate concentration in the NS (Figure 4). The relative representation of *SaNPF6.3* transcripts increased at low nitrate concentration (0.5 mM) and decreased at standard nitrate concentration (15 mM) as NaCl concentration increased (Figure 4a). Increasing the nitrate concentration up to 5 mM in the nitrate-deficient NS resulted in induction of the transporter expression (Figure 4b). Stimulation of the *SaNPF6.3* expression was also observed in response to salt shock (Figure 4c). The *SaNPF6.3* expression profiles calculated relative to those of single genes (*ACT7*, *TUA*) were different from profiles calculated based on normalization factors using two reference genes. The use of single reference genes for normalization of expression resulted in larger errors in the calculations (Figure 4).

## Discussion

To select the stably expressed genes in the *S. altissima*, we checked the housekeeping genes [43] which encode cytoskeleton proteins (SaACT7 actin and SaTUA α-tubulin), proteins related to protein synthesis (SaL2 ribosome large subunit protein, SaeEF1α translation elongation factor) and controlled protein degradation (SaUBC10 ubiquitin-conjugating protein) as well as proteins for presumably stable biochemical and regulation processes (SaGAPDH glyceraldehyde-3-phosphate dehydrogenase, SaPP2A protein phosphatase).

For analysis of the gene expression, we employed three algorithms implemented in GeNorm, NormFinder, and BestKeeper programs. The GeNorm algorithm is based on pairwise comparison of expression levels of several candidate genes and calculation of the corresponding variances. The main shortcoming of this method is the dependence of the results on the correlation in expression levels of different candidate genes, if the latter are subjected to co-regulation [45]. In the NormFinder program, the approach based on modeling the expression of each reference gene separately is used instead of pairwise comparison of gene expression levels. In this case, experimental samples are divided into several groups, and the measure of the candidate gene expression stability is the index, which is determined based on the calculation of intragroup and intergroup variances. Unlike the GeNorm method, the NormFinder algorithm is robust to the presence of correlation in the expression levels of co-regulated genes [46]. The BestKeeper algorithm, like geNorm, is based on pairwise comparison of the candidate gene expression levels, but includes correlation analysis of the expression of compared candidate gene pairs [47].

The stability of the pre-selected candidate gene expression differed markedly depending on the experimental conditions, i.e. the changes in ionic composition of the NS during the experimental procedures. For example, the transcript level of cytoskeleton *S. altissima ACT7* fluctuated when NaCl concentration changed in the NS in the long-term salinity experiments, but was maintained at the control level under salt-shock conditions.

Comparing the results of this study with *S. altissima* with those obtained using other plant species, expression stability of a particular gene depends on plant species. The gene *GAPDH* from *S. altissima* occupied a predominantly intermediate position in the expression stability series of the candidate genes; however, its ortholog from *S. aralocaspica* was one of the most stably expressed genes in a study of the effects of salinity [40]. High stability of *ACT7* expression was observed in *Arachis hypogaea* [48] and in a study of the effects of NaCl on *Apocynum venetum* [49], but an ortholog of this gene from *S. glauca* was one of the least stably expressed genes [42]. The cytoskeleton *TUA* gene was characterized by a stable expression in *S. glauca* [42], while according to the results of our work, the expression of its ortholog in *S. altissima* was either the least stable (when nitrate was added to nitrate-deficient medium and under salt shock) or intermediate (under long-term salinity conditions and different availability of nitrate). In the present study, the stability of protein synthesis/degradation *L2* and *UBC10* gene expression was investigated for the first time for a member of the genus *Suaeda*: *L2* was among the most stable genes under most treatments, especially in roots, *UBC10* had intermediate position.

Overall, our results confirm that it is not possible to select a universal reference gene suitable for normalizing the expression of target genes in different plants as well as studying the effects of different factors on the expression of a particular target gene. The reasons are likely in species-specific cell biology and gene expression patterns. The stability of expression for even the selected housekeeping genes can undergo considerable variation depending on the factors acting on the plant and is highly species-specific. Nevertheless, the results obtained in our work suggest that a number of these genes can be used to normalize the expression of *S. altissima* target genes in examining NaCl effects and differences in nitrate availability. For example, the gene of the ribosomal protein *L2* was one of the most stably expressed under almost all changes in the ionic composition of the NS of *S. altissima* plants in the course of our experiments. It should be noted that the slight differences in expression stability observed for the same genes using geNorm, NormFinder, and BestKeeper may be attributed to differences in the mathematics of the algorithms.

The changes in the expression of the selected housekeeping genes in plant organs and under different experimental treatments are themselves of interest. For example, the difference between Cq values for the cytoskeleton genes *SaACT7* actin and *SaTUA* α-tubulin was higher (in favor of *SaACT7* actin and its abundance over *SaTUA* mRNA) in flowers of 60-day-old plants (Supplementary materials, Table S3) posing questions about the abundance of the corresponding proteins and cytoskeleton rearrangements during development. Interestingly and oppositely, actin7 is a vegetative actin in *Arabidopsis* [50], while α1-tubulin gene of *A. thaliana* is preferentially expressed in flowers [51]. In this context, it is useful to compare results of quantitative real-time PCR with results from RNA sequencing and microarrays: each of the methods has shortcomings and errors bringing discrepancies between them – although housekeeping genes of actin and *GAPDH* expressed similarly in each method [52]. So far, the information on halophyte gene expression (in particular for species of the genus *Suaeda*) comes mostly from quantitative real-time PCR, while RNA sequencing has only been completed for a few species. For *Suaeda maritima*, the results of RNA sequencing demonstrated similar expression changes to qPCR for most transcription factors under salinity treatment [53]. Results of RNA sequencing also mostly coincided with qPCR for ten dysregulated genes checked in *Suaeda salsa* under 300 mM NaCl [54].

The opportunity of using the selected stable expressed genes of *S. altissima* and choosing the best way of normalization was tested for the nitrate transporter *SaNPF6.3*, a putative ortholog of the transporter/transceptor *AtNPF6.3* (*CHL1*/*NRT1.1*) [55] (Figure 4). We have previously demonstrated a nitrate-transporting function of *SaNPF6.3* and suggested that this protein may also be an element of the system involved in maintaining chloride homeostasis under salinity as well as processes underlying salt tolerance in *S. altissima* [27]. Normalization factor calculated on the basis of two or more reference genes has preference over using a single reference gene improving normalization reliability in RT-qPCR and decreasing its errors [45]. Our results confirm this conclusion. To determine the optimal number of reference genes for normalizing *SaNPF6.3* expression in RT-qPCR, geNorm program was used. The combination of two of the most stably expressed genes of the seven candidate genes was sufficient to normalize expression of this gene in *S. altissima* roots. The transcript representation of *SaNPF6.3* in roots under long-term salinity and after nitrate addition (Figure 4a,b, black bars) was determined relative to NF calculated from the expression levels of the two stably expressed genes, *SaPP2A* and *SaeEF1α*. In the case of salt shock, two other candidate genes were used for NF calculation, *L2* and *ACT7* (Figure 4c, black bars). For comparison, the *SaNPF6.3* transcript levels were also obtained using only one reference gene (Figures 4a–c, gray bars), *ACT7* for conditions of long-term salinity and nitrate addition and *TUA* for salt shock. The advantage of using two reference genes over a single one for the *SaNPF6.3* normalization can be also seen in this work with NFs on the basis of two genes (Figure 4a–c, black bars) and in our previous study [27] where *SaNPF6.3* transcript abundance was detected under the same conditions and with the same genes (*SaPP2A* or *SaeEF1α*, for normalization) but using them as single reference gene. With all experimental designs, similar results were obtained in the present and previous studies. It should be noted that a similar dependence of *SfNPF6.3* transcript abundance on NaCl concentration in the medium was also previously observed [31]. Although the results obtained with NFs calculated using two and a single reference gene are generally similar, the former have the advantage of lower scatter in the resulting data and smaller standard errors.

The set of genes used here and the study of their expression stability under different saline conditions and different availability of nitrate can serve as a basis for conducting similar experiments on other halophytes. The selected genes can serve as reference genes in RT-qPCR in studies of salt tolerance mechanisms of *S. altissima* and closely related species. The genes expressed at relatively low levels (*L2*, *TUA*, *PP2A*) can be used to normalize the expression of weakly expressed genes, such as genes of ion channels and transporters. The candidate genes with relatively high expression levels (*ACT7*, *eEF1α*, *GAPDH*) can be used as reference genes to investigate the expression for genes of interest with higher transcript representation, for example, genes of major metabolic pathways, such as photosynthesis and respiration, as well as genes involved in biosynthesis of osmotically active compounds and secondary metabolites.

## Supplementary Material

Supplemental Material

## Data Availability

The datasets used and/or analyzed during the current study are available from the corresponding authors upon reasonable request.
