# Supplementary material for: Identification and selection of reference genes for analysis of gene expression by quantitative real-time PCR in the euhalophyte Suaeda altissima (L.) Pall
Source: Commun Integr Biol. 2024 Jul 8;17(1):2372313. doi: 10.1080/19420889.2024.2372313 (PMC11236294; doi:10.1080/19420889.2024.2372313)
Supplement: Supplemental Material [file KCIB_A_2372313_SM2840.docx]

**Supplementary materials**

**Figure S1**

**
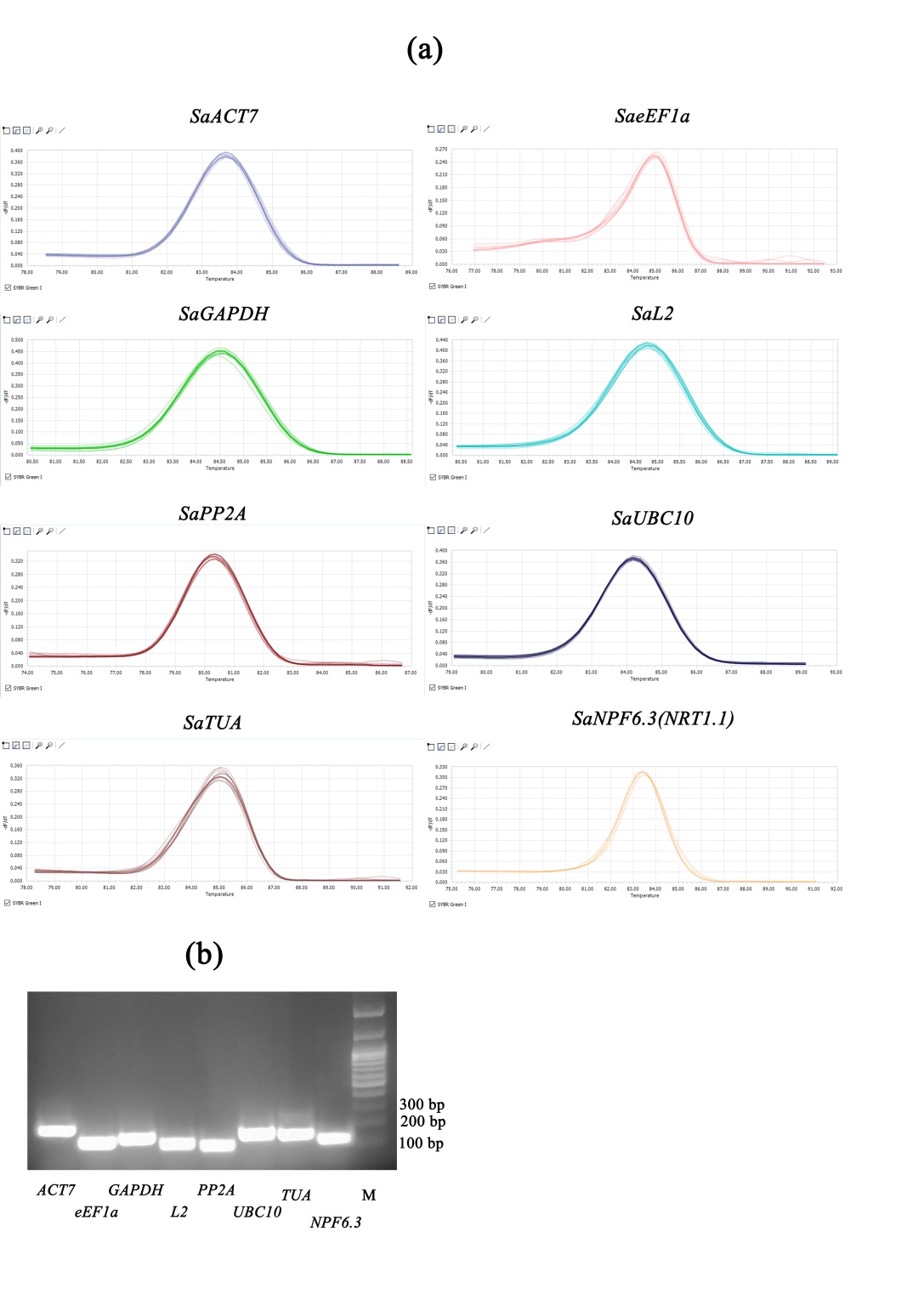
**

**Table S1.** List of primers used.

| Primer name | Sequence 5′-3′ | Application |
| --- | --- | --- |
| fullSaAct7_F | ATGGCTGATGCAGAGGAGATTC | Amplification of the full-length coding sequence of *SaACT7*, 1131 bp |
| fullSaAct7_R | CTTAGAAGCACTTTCGGTGAAC |  |
| SaeEF1a_F | ATGGGTAAGGAGAAGATTC | Amplification of the full-length coding sequence of *SaeEF1a*, 1317 bp |
| SaeEF1a_R | CTTCTTAAGGGCGGCCTTG |  |
| fGAPDH_F | ATGGCCAAGATTAAGATCGGAATCA | Amplification of the full-length coding sequence of *SaGAPDH*, 1011 bp |
| SfGAPDH_R | TTACTGGGTCTTTGACATGTGAACA |  |
| SfL2_F | ATGGGAAGAGTCATCCGAGCTCA | Amplification of the full-length coding sequence of *SaL2*, 780 bp |
| SfL2_R | TTAAGCCTTGTCGCTCTTGGAAAT |  |
| SfUBC10_F | ATGGCGTCGAAGAGAATCTTGAAG | Amplification of the coding sequence of *SaUBC10*, 444 bp |
| SfUBC10_R | TCAACCCATTGCATATTTCTGAGTC |  |
| SfTUA5_F | ATGAGAGAAATAATAAGCATACACATAGG | Amplification of the coding sequence of *SaTUA5*, 1245 bp |
| SfTUA5_R | TTCTTCCATGCCTTCACCAACATAC |  |
| SfPP2A_F | ATGCCGTCACACGCGGATCTAGAT | Amplification of the full-length coding sequence of *SaPP2A*, 918 bp |
| SfPP2A_R | TCACAAAAAATAGTCAGGGGTCTTG |  |
| SaeEF1alfa_F1 | TGAGATGTGTGGCAATCC | RT-qPCR, *SaeEF1a*, amplicon size – 128 bp |
| SaeEF1alfa_R1 | GTTGCTTCTGACTCCAAGAAT |  |
| SaAct7_F | AGATTCCGTTGCCCAG | RT-qPCR, *ACT7*, amplicon size –191 bp |
| SaAct7_R | ATTTCCTTGCTCATACGGTCA |  |
| SaGAPDH_RT_F1 | AATGGCTTTCCGTGTCCCAA | RT-qPCR, *GAPDH*, amplicon size – 149 bp |
| SaGAPDH_RT_R1 | CCTCGTCGGTGTAACCCAAA |  |
| SaPP2A_RT_F1 | GAGCTTTTTCGTATCGGCGG | RT-qPCR, *SaPP2A*, amplicon size – 103 bp |
| SaPP2A_RT_R1 | CCAGCAGCGTAACAGTCTCT |  |
| SaTUA_RT_F1 | GGCAAGGAGGATGCTGCTAA | RT-qPCR, *SaTUA*, amplicon size – 148 bp |
| SaTUA_RT_ R1 | CACCACCAACGGCATTGAAG |  |
| SaUBC10_RT_F1 | ACTTCGTGTAGTGCAGGTCC | RT-qPCR, *UBC10*, amplicon size – 159 bp |
| SaUBC10_RT_R1 | CGTCTTGAATGCCACCTTTGG |  |
| SaL2_RT_F1 | TAGTGGTTGCCGTGCTATGA | RT-qPCR, *SaL2*, amplicon size – 114 bp |
| SaL2_RT_R1 | GGCCAGCAGTTTCTCTTGAC |  |
| SaNRT1.1_F1 | CCTAGTCGAAATCAGGCG | RT-qPCR, *SaNPF6.3*, amplicon size – 116 bp |
| SaNRT1.1_R1 | CTCCCACGAAGAAGAATTGAG |  |

**Table S2.** Candidate reference genes of *S. altissima*.

| Gene | Number in the database  NCBI GenBank Data ID | Full name: SaACT7, SaUBC10 SaGAPDH glyceraldehyde-3-phosphate dehydrogenase, SaL2, SaTUA α-tubulin, SaeEF1α translation elongation factor, and SaPP2A protein phosphatase. | Percentage of identical amino acids | *A.thaliana* homolog, number in the TAIR database |
| --- | --- | --- | --- | --- |
| *SaAct7* | MK615596 | actin | 97% | AT5G09810 |
| *SaeEF1α* | MN076325 | translation elongation factor α | 92% | AT1G07940 |
| *SaGAPDH* | OP752353 | glyceraldehyde-3-phosphate dehydrogenase | 92% | AT1G13440.1 |
| *SaL2* | OP752354 | ribosome large subunit protein | 93% | AT2G18020.1 |
| *SaPP2A* | OP752355 | proteinphosphatase | 92% | AT1G69960 |
| *SaUBC10* | OP752356 | ubiquitin-conjugating protein | 96% | AT5G53300.1 |
| *SaTUA* | OP752357 | α-tubulin | 97% | AT5G19780.1 |

**Table S3.** Cq threshold cycle values of candidate reference genes in organs of 21-, 45-, and 60-day-old *S. altissima* plants.

| Gene | 21-day-old seedlings | | | | 45-day-old plants | | 60-day-old plants |
| --- | --- | --- | --- | --- | --- | --- | --- |
|  | leaf | cotyledon | stem | root | leaf | root | flowers |
| *SaAct7* | 18.50 ± 0.77 | 18.77 ± 0.82 | 17.68 ± 0.78 | 18.81 ± 0.14 | 16.61 ± 0.31 | 16.77 ± 0.83 | 19.62 ± 0.06 |
| *SaeEF1a* | 17.55 ±0.77 | 18.42 ± 0.44 | 17.36 ±0.37 | 17.98 ± 0.11 | 16.70 ± 0.19 | 15.69 ± 0.69 | 18.11 ± 0.19 |
| *SaGAPDH* | 18.31 ± 0.81 | 19.24 ± 0.19 | 18.80 ± 0.56 | 18.54 ± 0.67 | 17.55 ±0.48 | 18.02 ± 0.45 | 20.25 ± 0.17 |
| *SaL2* | 19.35 ± 0.81 | 20.70 ± 0.38 | 19.26 ±0.84 | 20.11 ± 0.39 | 18.60 ± 0.06 | 18.68 ± 0.45 | 20.21 ± 0.09 |
| *SaPP2A* | 23.86 ± 0.82 | 24.78 ± 0.64 | 23.89 ± 0.55 | 24.39 ±0.53 | 21.94 ± 0.24 | 21.67 ± 0.56 | 24.94 ± 0.02 |
| *SaTUA* | 21.53 ± 0.58 | 22.45 ± 0.55 | 21.26 ±0.34 | 21.96 ±0.48 | 20.88 ± 0.16 | 21.97 ± 0.84 | 25.45 ± 0.16 |
| *SaUBC10* | 19.46 ± 0.51 | 19.78 ± 0.67 | 18.66 ± 0.53 | 19.37 ± 0.27 | 17.66 ± 0.18 | 18.74 ± 0.63 | 20.95 ± 0.08 |

**Table S4.** Pairwise variation values (V_n_/_n+1_) of normalization factors of *S. altissima* candidate reference genes.

|  | V_2/3_ | V_3/4_ | V_4/5_ | V_5/6_ | V_6/7_ |
| --- | --- | --- | --- | --- | --- |
| Long-term salinity, root | 0.083 | 0.080 | 0.097 | 0.067 | 0.093 |
| Long-term salinity, leaf | 0.119 | 0.145 | 0.114 | 0.120 | 0.115 |
| Nitrate addition, root | 0.103 | 0.084 | 0.084 | 0.102 | 0.207 |
| Nitrate addition, leaf | 0.232 | 0.149 | 0.165 | 0.152 | 0.187 |
| Salt shock, root | 0.094 | 0.090 | 0.072 | 0.18 | 0.142 |
| Salt shock, leaf | 0.0824 | 0.0550 | 0.0425 | 0.0500 | 0.0423 |
